# First-line chemoimmunotherapy in metastatic breast carcinoma: combination of paclitaxel and IMP321 (LAG-3Ig) enhances immune responses and antitumor activity

**DOI:** 10.1186/1479-5876-8-71

**Published:** 2010-07-23

**Authors:** Chrystelle Brignone, Maya Gutierrez, Fawzia Mefti, Etienne Brain, Rosana Jarcau, Frédérique Cvitkovic, Nabil Bousetta, Jacques Medioni, Joseph Gligorov, Caroline Grygar, Manon Marcu, Frédéric Triebel

**Affiliations:** 1 Immutep S.A., (2 rue Jean Rostand), Orsay, (91893), France; 2 Centre René Huguenin, Saint Cloud, France; 3 Hôpital Européen Georges Pompidou, (20 rue Leblanc), Paris, (75908), France; 4 Hôpital Tenon, (4 rue de la Chine), Paris, (75970), France

## Abstract

**Background:**

IMP321 is a recombinant soluble LAG-3Ig fusion protein that binds to MHC class II with high avidity and mediates APC and then antigen-experienced memory CD8^+ ^T cell activation. We report clinical and biological results of a phase I/II in patients with metastatic breast carcinoma (MBC) receiving first-line paclitaxel weekly, 3 weeks out of 4.

**Methods:**

MBC patients were administered one dose of IMP321 s.c. every two weeks for a total of 24 weeks (12 injections). The repeated single doses were administered the day after chemotherapy at D2 and D16 of the 28-day cycles of paclitaxel (80 mg/m^2 ^at D1, D8 and D15, for 6 cycles). Blood samples were taken 13 days after the sixth and the twelfth IMP321 injections to determine sustained APC, NK and memory CD8 T cell responses.

**Results:**

Thirty MBC patients received IMP321 in three cohorts (doses: 0.25, 1.25 and 6.25 mg). IMP321 induced both a sustained increase in the number and activation of APC (monocytes and dendritic cells) and an increase in the percentage of NK and long-lived cytotoxic effector-memory CD8 T cells. Clinical benefit was observed for 90% of patients with only 3 progressors at 6 months. Also, the objective tumor response rate of 50% compared favorably to the 25% rate reported in the historical control group.

**Conclusions:**

The absence of toxicity and the demonstration of activity strongly support the future development of this agent for clinical use in combined first-line regimens.

**Trial registration:**

ClinicalTrials.gov NCT00349934

## Background

Traditionally, the goal of chemotherapy has been seen as direct cytotoxicity and induction of tumor cell death. However, the immunoadjuvant effect of chemotherapy, where the immune response induced by tumor cell death mediates the suppression of tumor growth and determines the long-term survival of patients, is now well established. For instance, in breast cancer patients who receive adjuvant chemotherapy, the analysis of metastasis-free survival showed an overall significantly lower percentage of metastasis-free patients in the group with mutated TLR4 [1]. The effect of the TLR4 mutation is to reduce antigen-presenting cell function. Such patients could not benefit fully from the immunological component of chemotherapy, i.e. the induction of cytotoxic CD8 T cell responses to tumor antigens released by dying tumor cells. A similar observation has been reported more recently in advanced colon cancer treated with oxaliplatin [2] and further supports the idea that apoptotic cell death induced by chemotherapy leads to a beneficial immunoadjuvant effect [1,3,4]. Enhancing such chemotherapy-induced T cell responses by giving a non-specific immunostimulatory factor which induces the antigen presenting cells (APCs) to mature and transport the tumor antigens to the lymph nodes for presentation to T cells would make such a combination therapy very attractive. This therapeutic approach is supported by preclinical studies which have shown synergy between chemotherapy and immunotherapy in carcinomas [5-8].

The soluble LAG-3Ig fusion protein (or IMP321) is a first-in-class immunopotentiator targeting MHC class II^+ ^APCs [9-12]. It has been tested in previously-treated advanced renal cell carcinoma patients known to be immunosuppressed and shown to induce an increase in the percentage of circulating activated CD8 T cells and of long-lived effector-memory CD8 T cells in all patients treated by repeated injections over 3 months, without any detectable toxicity [13]. Importantly, a concentration of only a few ng/mL IMP321 has been shown to be active *in vitro *on APC, showing the great potency of IMP321 as an agonist of the immune system [13].

In this report, data are presented demonstrating that IMP321 expanded and activated for several months both the primary target cells (MHC class II^+ ^monocytes/dendritic cells) to which IMP321 binds, and the secondary target cells (NK/CD8^+ ^effector memory T cells) which are activated subsequently. By pooling results from all 30 patients and comparing tumor regression with an appropriate historical control group, we saw a doubling of the objective response rate which suggests that IMP321 is a potent agonist of effective anti-cancer cellular immune responses in this clinical setting.

## Methods

### Patients

Patient characteristics are shown in Table 1. Eligible patients were at least 18 years old, had histologically documented stage IV breast adenocarcinoma, an Eastern Cooperative Oncology Group performance status of 0 or 1, measurable disease, adequate bone marrow, liver and renal function, and life expectancy of at least 3 months. Previous hormonal therapy for metastatic breast cancer or cytotoxic adjuvant chemotherapy was allowed. Biphosphonate therapy was allowed if started at least 4 weeks prior to first dosing of the study drug.

**Table 1:** Patients characteristics

Number of patients	n = 30
Years of age -- median (range)	64 (47-78)
Estrogen-receptor status -- no. (%)	
Positive	26 (87%)
Negative	4 (13%)
Progesterone-receptor status -- no. (%)	
Positive	18 (60%)
Negative	12 (40%)
HER2 status -- no. (%)	
Positive	0 (0%)
Negative	30 (100%)
Previous adjuvant chemotherapy -- no. (%)	
None	3 (10%)
Anthracycline	15 (50%)
Anthracycline + Taxane	8 (27%)
Vinorelbine	1 (3%)
Disease-free interval -- no. (%)	
≤ 24 mo	7 (23%)
> 24 mo	23 (77%)
Extent of disease -- no. (%)	
≥ 3 sites	22 (73%)
< 3 sites	8 (27%)
Location of disease -- no. (%)	
Visceral	21 (70.%)
Non Visceral	9 (30.%)
ECOG Status	
0	7 (23%)
1	22 (73%)

Patients were excluded if they were candidates for treatment with trastuzumab, had received prior chemotherapy for metastatic breast adenocarcinoma, or radiotherapy within the 30 days prior to first dosing of the study drug, known cerebral metastases or had a disease-free interval of less than 12 months from last dose of adjuvant chemotherapy.

Pregnant or nursing women were excluded. Women of childbearing potential were required to have a negative pregnancy test within 7 days of treatment initiation and to use adequate birth control measures during the study. Patients were excluded if they had severe allergy, known clinically active autoimmune disease requiring immunosuppressive therapy, known active hepatitis B or C, known HIV positivity, or had any condition that was unstable or could jeopardize their safety or ability to comply with study procedures, or could interfere with evaluation of the results. All patients gave written, informed consent to participate in the study, which was conducted in accordance with the Declaration of Helsinki, the Good Clinical Practice guidelines, and all applicable local laws and regulations. The study protocol and amendments were approved by an institutional review board and an independent ethics committee.

### Study design and treatments

**Figure 1:**
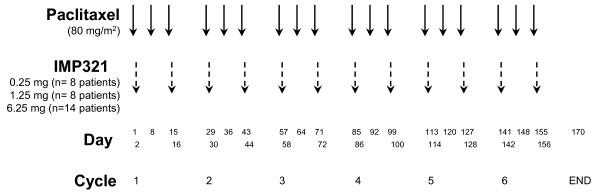
**First-line chemo-immunotherapy: drug administration schedule**. The repeated single doses of IMP321 (0.25, 1.25 and 6.25 mg s.c. 12 × q14) were administered on D2 and D16 of the 28-day paclitaxel cycle, i.e. on the day after chemotherapy. A fixed dose of paclitaxel (80 mg/m^2^) was given as a weekly, 3 weeks out of 4, chemotherapy regimen for 6 cycles.

In this multi-center open-label, non-randomized, fixed dose-escalation phase I/II trial performed in an ambulatory and day-hospital setting, patients were administered one dose of IMP321 s.c. every two weeks for a total of 24 weeks (12 injections in total). The repeated single doses were administered on D2 and D16 of each 28-day cycle of paclitaxel (80 mg/m^2 ^of paclitaxel via 1-hour infusion at D1, D8 and D15 of every 28-day cycle for 6 cycles), i.e. on the day after chemotherapy (Fig.1). Twenty mg i.v. dexamethazone were given in the first cycle before each paclitaxel infusion. Corticosteroids were not administered after the first chemotherapy cycle if the first 3 i.v. infusions of paclitaxel were well tolerated.

Eight to fourteen patients were enrolled in successive cohorts with the following IMP321 dosing: 0.25 mg, 1.25 mg and 6.25 mg per injection (Fig.1). To be evaluable for the decision to proceed with the next cohort at a higher dose level, a patient must have received at least 12 weeks of treatment with IMP321. Toxicities were assessed using the National Cancer Institute Common Toxicity Criteria version 3.0. Dose-limiting toxicity (DLT) was defined as any grade 3-4 toxicity. If one patient had developed a DLT, dose escalation would have been stopped and the prior dose level considered the maximum tolerated dose (MTD). No intra-patient dose escalation was permitted. Also, because of the fixed-dose study design no dose reduction for a patient was allowed.

### Study assessments

Before initiating treatment, each patient was evaluated for medical history, physical examination, tumor measurement using computer-assisted tomography, Eastern Cooperative Oncology Group performance status, complete differential blood count, serum chemistry, urinalysis, and electrocardiogram. These assessments were also done before each subsequent injection. All observations were recorded, including results of physical examinations, vital signs, adverse events, concomitant medications, and laboratory tests. Patients were monitored every 2 weeks and as needed for adverse events. Tumor response and progression were assessed using Response Evaluation Criteria in Solid Tumors (RECIST) version 1.1 [14] with imaging studies done 2 weeks after the sixth and the twelfth injections. Sera were collected at baseline (D1) and two weeks after the twelfth (day 170) injection for detection of serum anti-IMP321 antibodies. Blood samples were collected in lithium heparin-containing tubes at the same time points and also two weeks after the sixth injection (day 85) for monitoring both the APC (monocytes, dendritic cells) and CD8 T cell immune responses.

### Detection of anti-IMP321 antibodies

Serum samples obtained at baseline and day 170 after the initial dosing were tested for antidrug antibodies using ELISA. The serum was diluted 1:100 to avoid matrix effect, loaded (at least two determinations/sample) on microtiter plates (Maxisorb, NUNC) precoated with IMP321 (1 μg/well) and revealed by a mix of HRP-conjugated goat anti-human kappa and goat anti-human lambda antibodies (Serotec). As controls, various concentrations of a recombinant human monoclonal antibody fragment Fab-dHLX-MH directed to IMP321 produced from human Ig library in E. coli (MorphoSys, Martinsried, Germany) were added to each plate and the assay sensitivity was 3 ng/ml Fab equivalent. Optical densities (OD) were determined at the wavelengths of 450 and 600 nm.

The sera from some patients were also assessed in a bridging immunogenicity assay using the electrochemiluminescence Meso Scale Discovery (MSD) analyzer [15]. Briefly, any drug-specific antibodies present in undiluted serum samples were captured and revealed by biotin- and SULFO-Tag-conjugated IMP321 respectively, on streptavidin-coated plates. An anti-LAG-3 mAb (17B4) diluted in neat human AB (Jacques Boy) was used as reference standard (see [13] for details).

### Pharmacodynamics

Blood samples were collected pre-dosing at D1, D85 and D170 and directly stained with BD Multitest CD8-FITC/CD38-PE/CD3-PerCP/HLA-DR-APC, with BD Multitest 6-color TBNK (CD3-FITC/CD16-PE+CD56-PE/CD45-PerCPCy5.5/CD4PE-Cy7/CD19-APC/CD8-APC-Cy7), BD Simultest LeucoGate (CD45-FITC/CD14-PE) in tubes containing a precise number of fluorescent control beads (BD Trucount™tubes, BD Biosciences). After lysis of red blood cells using the BD FACS lysing solution, cells were analyzed using a 6-color FACSCanto cytometer and the absolute number of cells per μl of whole blood was calculated using the number of control beads acquired in each sample. For patients in the 1.25 and 6.25 mg groups, cells were also directly stained in BD Trucount™with CD45-APC-Cy7, CD14-PerCP, anti-HLA-DR-PE-Cy7 and different combinations of monocyte activation markers: CD11a-FITC, CD11b-PE, CD16-PE, CD35-FITC, CD54-PE, CD64-FITC, CD80-PE and CD86-FITC. The expression of these markers on monocytes was directly proportional to the cell-bound fluorescence. The results are shown after normalization of the cell-bound fluorescence against the fluorescence of control beads.

PBMCs were isolated by centrifugation over Ficoll-Paque (GE Healthcare) using LeucoSep^® ^tubes (Greiner Bio-One). Fresh PBMCs were stained with CD45-FITC, CD14-PerCP, CD16-PE-Cy7, HLA-DR-APC-Cy7, CD11c-APC and CD123-PE antibodies to determine the percentage of plasmacytoid (pDC, CD45^+ ^CD14^- ^CD16^- ^HLA-DR^+ ^CD123^+ ^CD11c^-^) and myeloid (mDC, CD45^+ ^CD14^- ^CD16^- ^HLA-DR^+ ^CD123^- ^CD11c^+^) dendritic cells in CD45^+ ^cells. Cells (0.3-1.0 × 10^6^) were washed in Dulbecco's phosphate-buffered saline (Invitrogen) 0.5% bovine serum albumin (PAA Laboratories), 0.1% sodium azide (Sigma-Aldrich), and incubated with the mixture of fluorochrome-conjugated antibodies (all from BD Biosciences) for 30 min at 4°C. After washing, cells were analyzed by cytometry. The remaining cells were frozen in fetal calf serum (Hyclone) supplemented with 10% DMSO (Sigma-Aldrich). Following completion of the protocol, series of PBMC samples for each individual were thawed and stained with CD3-PerCP-Cy5.5, CD8-APC-Cy7, CD45RA-PE-Cy7, CD45RO-APC and CD62L-FITC antibodies to determine the percentage of CD8^+ ^T cells (secondary target cells) with a terminally differentiated CD45RA^+ ^effector memory (EM) phenotype. These EMRA cells are CD3^+ ^CD8^+ ^CD45RA^+ ^CD45RO^- ^CD62L^-^.

### Statistical analysis

The paired non-parametric Wilcoxon signed rank test was used to compare the immunomonitoring and clinical values obtained at the different time points. Only patients with all available time points were included in the immunomonitoring analysis. Spearman rank correlation coefficients were estimated for bivariate analyses. The *a priori *level of significance was a p-value of < 0.05. Data was computed using JMP^® ^software.

## Results

### Safety

Two out of the 33 enrolled patients were removed from the 1.25 mg arm of the study early on and were replaced because of persistent grade 3 paclitaxel-related neuropathy. A third case received one injection IMP321 (6.25 mg) and was removed from the study due to ineligibility. All the other 30 patients received at least 6 doses of IMP321 and were included in both the safety and efficacy analyses. No clinically significant local or systemic IMP321-related adverse events were recorded, in line with a previous study in which IMP321 was used alone (i.e. without chemotherapy) [13]. With the combination therapy, six grade 3 adverse events were recorded (four in the 0.25 mg group and one in the 1.25 mg and 6.25 mg group each): asthenia (3 cases), neuropathy, allergic reaction and neutropenia. In addition, one patient in the 0.25 mg group had appendicitis, one patient in the 1.25 mg group a *Staphylococcus *infection and one patient in the 6.25 mg group an accidental hip bone fracture.

### Detection of anti-IMP321 antibodies

Sera collected at baseline and two weeks after the twelfth injection (day 170) were assayed for anti-IMP321 antibodies by direct ELISA (Additional file 1). Two patients receiving 1.25 mg (patients #11 and #12) showed an increase compared to baseline level by more than 15%. The sera from these (and other) patients were then assayed without any dilution in a very sensitive bridging immunogenicity assay using the MSD analyzer [15]. The tested sera gave a signal below the detection range (2 ng/ml). Repeated injection of IMP321 up to 6.25 mg did not induce anti-IMP321 antibodies.

### Pharmacodynamics

**Figure 2:**
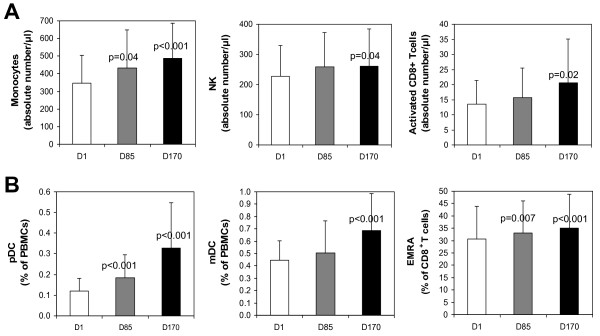
**IMP321 increases the numbers of monocytes, NK and activated CD8 T cells in blood (panel A)**. Fresh blood samples were collected pre-dosing, at D1, D85 and D170 and directly stained with fluorochrome-conjugated antibodies in tubes containing a precise number of fluorescent control beads. The results show the mean ± sd of the absolute numbers of CD45^+^CD14^+ ^(monocytes), CD3^-^CD56^+ ^(NK cells) and CD38^+^HLA-DR^+^CD8^+ ^(activated CD8^+ ^cells) cells. The paired non-parametric Wilcoxon signed rank test was used to compare increases observed between D85 or D170 and D1. When significant (< 0.05), p values are indicated. ***IMP321 increases the percentages of dendritic cells and cytotoxic CD45RA^+ ^Effector-Memory CD8^+ ^T cells (EMRA) in PBMCs (panel B). ***PBMCs cells collected pre-dosing, at D1, D85 and D170 were isolated and stained with fluorochrome-conjugated antibodies and analyzed by flow cytometry. The results show the mean ± sd of the percentages of plasmacytoid dendritic cells (pDC, CD45^+^CD14^-^CD16^-^HLA-DR^+^CD123^+^CD11c^-^) and myeloid dendritic cells (mDC, CD45^+^CD14^-^CD16^-^HLA-DR^+^CD123^-^CD11c^+^) in CD45^+ ^cells, as well as the percentages of CD45RA^+^CD45RO^-^CD62L^- ^in the CD8^+ ^T cell population (CD45RA^+^EM CD8 T cells or EMRA). The significant Wilcoxon p values are indicated.

Blood samples were tested 13 days after each IMP321 injection (i.e. just before the chemotherapy and 24 hr before the next IMP321 injection): therefore only sustained cell activation or cell subset expansion was analyzed. Immunomonitoring in fresh whole blood showed significant increases of monocytes (CD45^+^CD14^+^, 20 patients out of 24), NK cells, (CD3^-^CD16^+^CD56^+^, 15 out of 24) and activated (CD38^+^HLA-DR^+^) CD8 T cells (17 out of 24) in absolute numbers per μl of fresh whole blood on D170 compared to D1 (Fig. 2 panel A).

**Figure 3:**
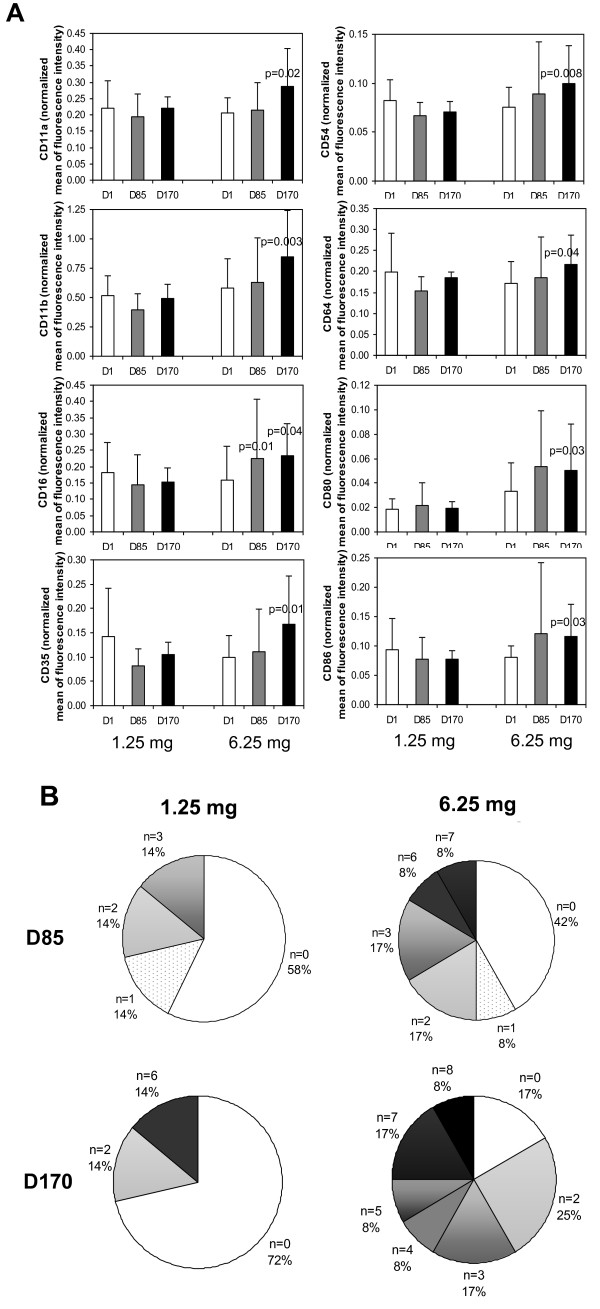
**IMP321 increases the expression of activation markers on blood monocytes**. Blood samples were collected pre-dosing, at D1, D85 and D170 and directly stained with fluorochrome-conjugated CD45, CD14, anti-HLA-DR and CD11a, CD11b, CD16, CD35, CD54, CD64, CD80 or CD86 antibodies in tubes containing a precise number of fluorescent control beads. The expression of activation markers on monocytes was directly proportional to the cell-bound fluorescence. The results shown in panel A are the mean ± sd after normalization of the cell-bound fluorescence against the fluorescence of control beads. Statistically significant increases between D85 or D170 and D1 are analyzed using Wilcoxon signed rank test and significant p values (< 0.05) are shown. In panel B, the percentage of patients showing increases in the expression of the indicated numbers of activation markers at D85 or D170 compared to the baseline at D1 was calculated. The number of markers (n) displaying an increase by at least 50% was calculated for each patient in the 1.25 mg (7 patients) and 6.25 mg (12 patients) groups. The pie charts represent the percentages of patients with increases in the indicated number of markers.

Increases in the expression on blood monocytes of adhesion molecules (CD11a, CD11b, CD54), receptors for immunoglobulins (CD16 and CD64), complement (CD35) and co-stimulation molecules (CD80 and CD86) were consistently observed in patients receiving the 6.25 mg but not the 1.25 mg dose (Fig. 3A). To assess the functionality of monocytes after treatment, we considered the upregulation of the expression of any one of these 8 independent markers by more than 50% to be a gain of function. In the 1.25 mg-group, 28% of patients expressed a gain of function at D170 compared to D1 while 83% of patients in the 6.25 mg group did so (Fig. 3B). In addition, the gain of function was much more pronounced in the latter group as more than 50% of these patients upregulated at least one marker at D85 and 3 markers at D170 (Fig. 3B). The scores were calculated by multiplying the percentage of patients by the number of markers to apply more weight to patients presenting an increase in several activation markers. When the weighted scores of the two groups are compared using the non-parametric Wilcoxon test, we observed a greater activation index for the 6.25 mg-group than for the lower dose groups with borderline statistical significance (p = 0.06 at D170).

Immunomonitoring of PBMCs showed an increase in the percentages of plasmacytoid and monocytoid dendritic cells (pDC, 24 patients out of 25, mDC, 21 out of 25) and CD62L^-^CD45RA^+ ^effector-memory (EMRA, 21 patients out of 26) CD8 T cells which represent the most differentiated type of CD8^+ ^T cells (Fig. 2 panel B)[16,17].

In conclusion, we have seen an effect on:

- primary target cells (i.e. MHC class II^+ ^cells): the treatment increased the absolute numbers in whole blood as well as the percentages in PBMCs of APC (monocytes and dendritic cells). Monocytes were more activated at the higher IMP321 dose level for at least 3 months (i.e. from D85 to D170).

- secondary target cells: the treatment increased the absolute numbers of NK and activated CD8 T cells per μl of fresh whole blood as well as the percentage of CD8^+ ^T cells with a terminally differentiated effector memory phenotype in PBMCs. These CD8 T and NK subsets are known to display a high anti-tumor activity.

### Efficacy

**Figure 4:**
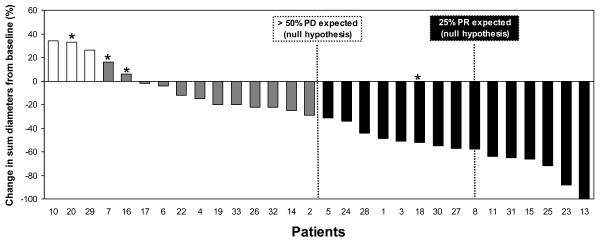
**Clinical results: comparison with historical control group**. The waterfall plot presents the percentage of change in the sum of tumor diameters observed after treatment (6 months) for individual patients. The patients experiencing progressive disease (PD), stable disease (SD), or partial response (PR) are shown in white, grey and black, respectively. Patients 1-8 received 0.25 mg, patients 9-18 1.25 mg and patients 19-33 6.25 mg IMP321. Asterisks show the 4 patients with 3 months treatment instead of 6 months. Historical data obtained for a group receiving paclitaxel alone are presented as dotted lines.

According to the protocol, 30 patients were retained for analysis out of the 33 enrolled patients. The three drop-out patients received just one or a few IMP321 injections (see Safety). The percentage change in the sum of tumor diameters at the end of treatment are shown in Fig. 4 as a waterfall plot. Only 3 out of 30 patients had progressive disease (10%) and 15 benefited from an objective tumor response (50%).

The historical control group is derived from the ECOG 2100 study, the only randomized phase III study with a very similar chemotherapy administration schedule using 90 mg/m^2 ^paclitaxel given on days 1, 8 and 15 every 4 weeks until disease progression [18]. In the subgroup of patients with measurable disease at inclusion (like our patients) the response rate was 25% (64 patients with partial or complete response out of 254). Progression-free survival (PFS) in the historical control group was only 5.6 months and therefore more than 50% of the patients were classified at 6 months as having progressive disease (PD, Fig. 4). In our case, only 3 patients out of 30 (i.e. 10%) were PD. Similarly, the objective response rate in the historical control group of 25% can be compared to our response rate of 15 patients out of 30 treated (50%) (Fig. 4).

**Figure 5:**
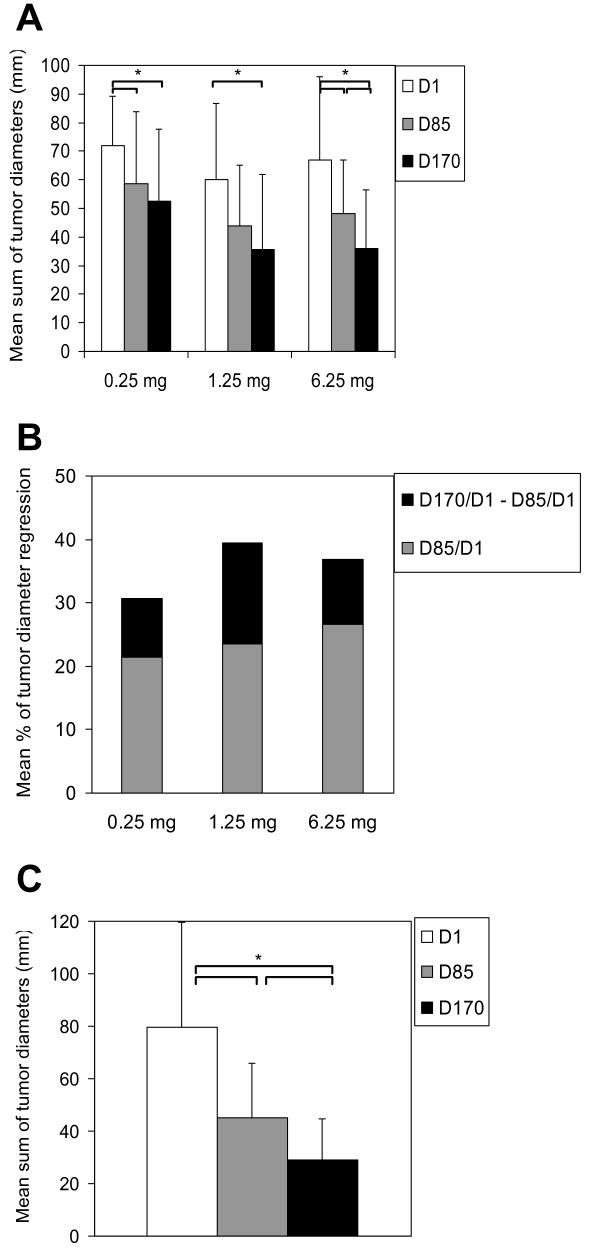
**Tumor regression during maintenance chemotherapy**. Mean sums of tumor diameters (panel A) and percentages of tumor regression (panel B) measured at inclusion (D1) and after 3 months (D85, i.e. during induction chemotherapy) and 6 months (D170, i.e. during maintenance chemotherapy) are shown for each dose-group. The four patients with unavailable D170 data were excluded from the analysis. Statistically significant decreases between D85 or D170 and D1 and between D170 and D85 are indicated (see brackets, * p < 0.05). The mean sums of tumor diameters ± sd obtained in the 15 patients who achieved an objective response are shown in panel C (one D170 sum of diameters is missing).

Closer analysis of the response at the different time-points reveals a particularly interesting difference in the time-profile of the response compared to paclitaxel alone. With chemotherapy alone, most of the tumor regression is usually observed during the first 3 months. This induction phase corresponds here to the period from D1 to D85. The changes in mean sum of tumor diameters and in mean percentage of tumor diameter regression for patients with available D85 and D170 target lesion measurements are shown in Fig. 5 panel A and panel B, respectively. In contrast to chemotherapy alone, further significant tumor regression was observed during the maintenance phase of the treatment (i.e. D85-D170), especially at the highest IMP321 dose (6.25 mg) where statistical significance was reached.

The mean tumor diameter regression percentage calculated on the 15 PR (Fig.5, panel C) was 40% in the first 3 months (i.e. induction chemotherapy between D1 and D85) in patients with an objective clinical response and a further 29% (i.e. D170 versus D85) in the next 3 months (i.e. maintenance chemotherapy between D85 and D170). In volumetric terms this corresponds to a shrinkage of 74% in the first three months (i.e. D85 versus D1) followed by a further 50% in the second three months (i.e. D170 versus D85).

Moreover, none of the patients receiving the lowest dose (0.25 mg) of IMP321, and experiencing an objective response after 6 months of treatment, had an objective tumor regression between D85 and D170. In contrast, 50% and 71% of patients in the 1.25 mg and the 6.25 mg groups, respectively, had a further objective clinical response during the last 3 months (data not shown).

**Figure 6:**
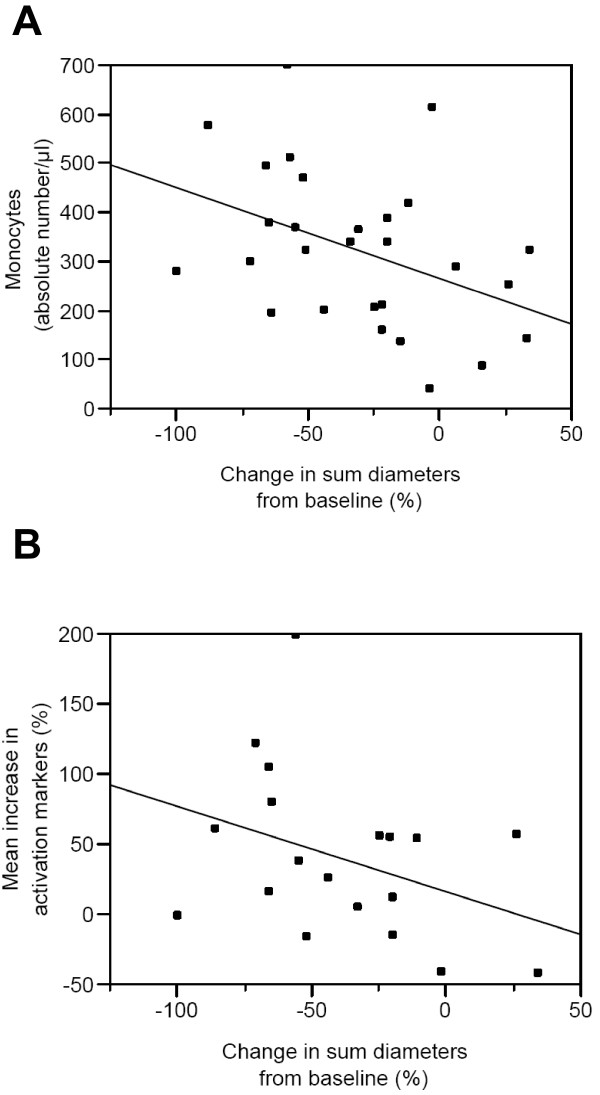
**Correlation between the number of monocytes before treatment and tumor regression (Panel A)**. The absolute numbers of monocytes per μl of blood at D1 are plotted as a function of the percentages of change in the mean sum of tumor diameters at D170 versus D1 (correlation coefficient ρ = -0.4; p < 0.05). ***Correlation between the monocyte gain of function at D170 and tumor regression (Panel B)***. The monocyte gain of function calculated as the mean of the percentage increases of each of the eight activation markers in Figure 3A is plotted as a function of the percentages of change in the mean sum of tumor diameters at D170 versus D1 (correlation coefficient ρ = -0.44; p = 0.05).

The change in tumor size (mean sum of tumor diameters at post-study relative to pre-study) is significantly correlated (Spearman rank correlation coefficient ρ = -0.4) with the absolute number of monocytes (CD45^+^CD14^+^) per μl of blood at D1 (Fig. 6A). Note that the normal range for monocytes is 0.3 - 0.8 × 10^9 ^CD45^+^CD14^+ ^cells/l whole blood [19] and therefore many patients and especially the poor responders are monocytopenic at D1.

We also analyzed whether there was a difference between responders and non-responders in the 1.25 and 6.25 mg groups in terms of monocyte gain of function, as defined in Fig.3B. The change in tumor size is significantly correlated (Spearman rank correlation coefficient ρ = -0.44) with the monocyte gain of function at D170 (Fig. 6B). There was no other significant correlation with regards to NK or CD8 subsets.

## Discussion

First-line chemo-immunotherapy is a new approach to the treatment of advanced cancer. Chemotherapy drugs alone induce tumor cell apoptosis and cause modulation of the immunological environment combined with a burst of tumor antigen release. The resulting T cell immune response contributes to the regression of the tumor [20]. However, this initial immune response needs to be prolonged and amplified by a T-cell booster that is non-toxic and can be given repeatedly, such as IMP321. IMP321 has a direct effect on APC which express MHC class II giving rise to rapid APC activation and leading to reactivation and expansion of antigen-experienced memory CD8 T cells [11].

In the present study, the change in tumor size is correlated with the absolute number of monocytes per μl of blood at D1 (Fig. 6). Notably, poor responders tend to be monocytopenic. Monocytes are the most common MHC class II^+ ^primary target cells for IMP321 in the blood [12] and therefore it makes sense that a higher number of monocytes before treatment should favor the tumor response to IMP321

We used the weekly, 3 weeks out of 4, chemotherapy regimen which was introduced to reduce cumulative neurotoxicity observed with weekly paclitaxel administration [18]. Repeated single doses of IMP321 were administered on D2 and D16 of the 28-day paclitaxel cycle, on the day after the chemotherapy, to activate the antigen-loaded APC. This repeated dose injection protocol has been shown separately to be well tolerated for doses up to 30 mg in advanced cancer patients and to induce CD8 memory T cell expansion [13].

In the present study, clinical benefit at 6 months was observed for 90% of patients in contrast to less than 50% of patients (PFS = 5.6 months) in the historical control group [18]. Also the objective response rate of 50% at the post study visit compared favorably to the 25% response rate in the historical control group [18]. Although the inclusion and exclusion criteria were similar, the resulting patient populations were not identical in the two studies. For instance we enrolled older patients (64 years old compared to 55) and more patients with extent of disease ≥ 3 sites (73% compared to 46%, p = 0.007). Future randomized clinical studies with an internal paclitaxel alone control group will determine whether the differences observed in the present study in terms of clinical benefit hold true.

The clinical benefit of immunotherapy may only appear after a few months of treatment as it takes time for active immunotherapy to progressively reinforce the immune response. For example, in patients treated with ipilimumab, an anti-CTLA-4 neutralizing antibody, the patterns of clinical response differ from those seen following cytotoxic chemotherapy. Late responses are frequent and may even occur after an initial period of tumor progression [21]. In the present study, for the 15 PR, the tumor regression during maintenance chemotherapy between D85 and D170 was not much less than that seen during induction chemotherapy between D1 and D85. This level of maintained response may be a characteristic and a benefit of chemo-immunotherapy protocols.

To investigate the role of the immune response behind the above clinical results, we analyzed both the absolute numbers of PBMC subsets and any changes in the proportions of key constituents. On top of a significant increase in the absolute number of monocytes, NK cells and activated CD8 T cells, we observed an increase in the proportion of the EMRA CD8^+ ^T cell subset. The importance of this subset in cancer was highlighted when it was shown for the first time that circulating EMRA cells exert *ex vivo *tumor-specific cytolytic activity in melanoma patients [22]. These EMRA cells have been characterized as terminally differentiated CD8 T cells potentially able to home into inflamed tissues such as the tumor microenvironment because they have lost the CD62L and CCR7 lymphoid homing receptors [16,17].

Memory T cells are generated and stored in secondary lymphoid organs, such as lymph nodes, spleen and the bone marrow [23]. There is a high frequency of memory T cells against breast tumor-associated antigen MHC class I-restricted peptides in the bone marrow of breast cancer patients [24]. Given that it is only after several rounds of proliferation that memory T cells appear in the peripheral blood, it may well be that the significant and sustained increase in EMRA CD8 T cells in the blood of our patients is an indication of a much greater expansion in the secondary lymphoid organs.

Importantly, EMRA cells are one of the long-lived T cell subsets. We have shown that a long-lived EM subset was increased by IMP321 in renal cell carcinoma patients [13]. Successful active immunotherapy may depend not upon a transient increase in short-lived effector T cells but rather upon the progressive reinforcement of these vital long-lived effector-memory subsets. If this is true it could explain why successful active immunotherapies exert their effects over years [25].

Although the anti-cancer mechanism of action of paclitaxel is initially due to effects on β-tubulin, growing evidence supports anti-tumor effects through innate immune activation and possibly through TLR4/MD-2 activation. In the mouse, paclitaxel can mimic bacterial LPS by activating macrophages and DC [26]. However this action of paclitaxel on mouse MD-2/TLR4 is in contrast with the observation that paclitaxel associates with human MD-2 *in vitro *without promoting TLR4 activation [27].

In patients, there is also circumstantial evidence that paclitaxel can stimulate the immune response against cancer. A clinical study indicated that the development of a lymphocytic infiltrate after chemotherapy correlated with a positive response to neoadjuvant paclitaxel therapy [3]. Like many cytotoxic compounds, paclitaxel may have an immuno-adjuvant effect that relies on the capacity of APC to engulf dying tumor cells and then process and present tumor antigens to memory T cells [20]. However, analyses of PBMC subsets in patients receiving taxane therapy have not revealed any alterations in these subsets in terms of percentages or phenotypes [28,29].

The 6.25 mg IMP321 dose was found in the present study to activate monocytes when given repeatedly over 6 months (see Fig.3). The gain of function in this major APC subset analyzed in fresh whole blood (fresh because monocytes are known to be very sensitive to freezing/thawing) was already observed at D85 and then strongly reinforced at D170. This gradual but sustained activation was observed in a circulating APC population expanded both in terms of absolute numbers per μl of blood and in terms of percentages of PBMCs. Among the different activation antigens analyzed, CD54 expression was increased. CD54 upregulation is currently used as a surrogate for assessing human APC activation and also as a potency measure of sipuleucel-T, an approved active cellular immunotherapy product designed to stimulate an immune response against prostate cancer [25,30]. These observations, combined with the lack of any IMP321 side effects, clearly indicate that a 6 mg IMP321 s.c. dose given biweekly for a long period of time will be a potent administration scheme for pivotal chemo-immunotherapy trials. We have shown separately that increasing the dose from 6 to 30 mg does not seem to increase the immune response in cancer patients [13].

## Conclusions

The next step will be to test immunotherapy combined with chemotherapy in a first-line setting with patients with a good immune status, as here, but in randomized phase II and III clinical trials with standard chemotherapy as a control arm. If successful this will confirm the idea that chemotherapy and immunotherapy can form a practical partnership in the treatment of cancer.

## Competing interests

CB, CG, MM and FT received salaries from Immutep S.A. which holds patents relating to the content of the manuscript.

## Authors' contributions

MG, FM, EB, RJ, FC, JM and JG carried out the clinical study. NB helped collect the data. CB, CG and MM carried out the immunoassays. CB participated in the design of the study and performed the statistical analysis. MG and FT conceived the study, and participated in its design and coordination and helped draft the manuscript. All authors read and approved the final manuscript.

## Supplementary Material

Additional file 1 **Anti-IMP321 antibodies**. Sera collected at baseline and 2 weeks after the sixth and the twelve IMP321 injections were tested for the presence of anti-IMP321 antibodies by direct ELISA. Absorbance values corresponding to various concentrations of an anti-IMP321 recombinant human Fab antibody fragment (left panel) are indicated. Click here for file
