# Supplementary figures and images for: First-line chemoimmunotherapy in metastatic breast carcinoma: combination of paclitaxel and IMP321 (LAG-3Ig) enhances immune responses and antitumor activity
Source: J Transl Med. 2010 Jul 23;8:71. doi: 10.1186/1479-5876-8-71 (PMC2920252; doi:10.1186/1479-5876-8-71)

## Slide 1
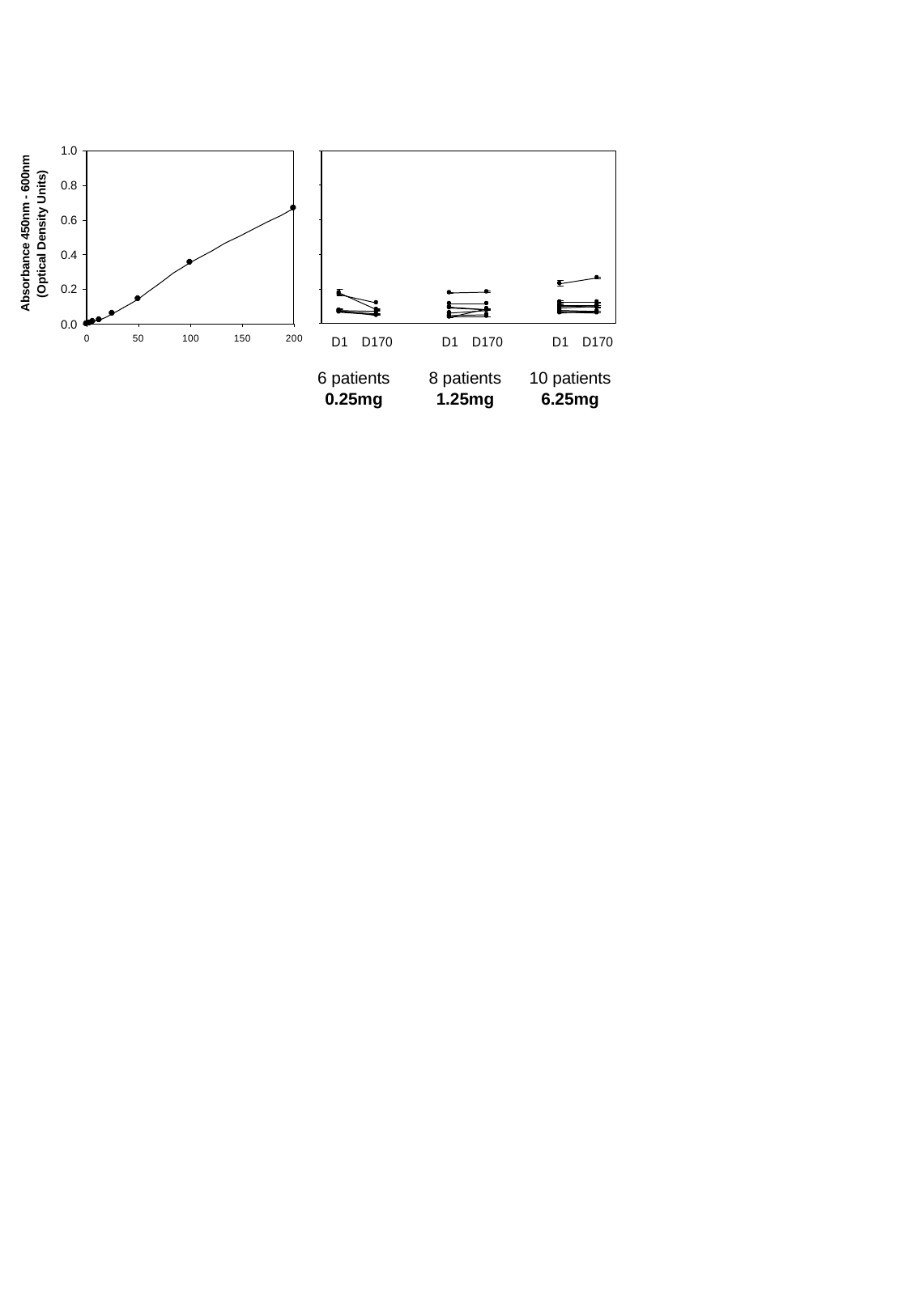

6 patients
0.25mg
8 patients
1.25mg
10 patients
6.25mg

Supplement: Additional file 1 — Anti-IMP321 antibodies. Sera collected at baseline and 2 weeks after the sixth and the twelve IMP321 injections were tested for the presence of anti-IMP321 antibodies by direct ELISA. Absorbance values corresponding to various concentrations of an anti-IMP321 recombinant human Fab antibody fragment (left panel) are indicated. [file 1479-5876-8-71-S1.PPT]
